# Association between Methylenetetrahydrofolate Reductase (MTHFR) Gene Polymorphisms and Susceptibility to Childhood Acute Lymphoblastic Leukemia in an Iranian Population

**Published:** 2016-07-01

**Authors:** Gholamreza Bahari, Mohammad Hashemi, Majid Naderi, Mohsen Taheri

**Affiliations:** 1 Cellular and Molecular Research Center, Zahedan University of Medical Sciences, Zahedan, Iran; 2 Department of Clinical Biochemistry, School of Medicine, Zahedan University of Medical Sciences, Zahedan, Iran; 3 Genetics of Non-Communicable Disease Research Center, Zahedan University of Medical Sciences, Zahedan, Iran

**Keywords:** MTHFR, Polymorphism, Acute lymphocytic leukemia

## Abstract

**Background:** The present study was aimed to examine the possible association between methylene tetrahydrofolate reductase (MTHFR) gene polymorphisms and childhood acute lymphoblastic leukemia (ALL) in a sample of Iranian population.

**Subjects and Methods:** A total of 220 subjects including 100 children diagnosed with ALL and 120 healthy children participated in the case-control study. The single nucleotide polymorphisms (SNPs) of MTHFR were determined by ARMS-PCR or PCR-RFLP method.

**Results:** Our investigation revealed that rs13306561 both TC and TC + CC genotypes decreased the risk of ALL compared to TT genotype (OR=0.32, 95%CI=0.15-0.68, p=0.002 and OR=0.35, 95%CI=0.17-0.70, p=0.003, respectively). In addition, the rs13306561 C allele decreased the risk of ALL in comparison with T allele (OR=0.42, 95% CI=0.22-0.78, P=0.005). MTHFR rs1801131 (A1298C) polymorphism showed that the AC heterozygous genotype decreased the risk of ALL in comparison with AA homozygous genotype (OR=0.43, 95%CI=0.21-0.90, p=0.037). Neither the overall Chi-square comparison of cases and control subjects (𝜒2=5.54, p=0.063) nor the logistic regression analysis showed significant association between C677T polymorphism and ALL (OR=1.25, 95% CI=0.69-2.23, p=0.552; CT vs. CC).

**Conclusion:** The current investigation findings showed that MTHFR rs1801131 and rs13306561 polymorphisms decreased the risk of ALL in the population which has been studied. Further studies with larger sample sizes and different ethnicities are required to validate our findings.

## Introduction

 Pediatric cancer is currently the second most common cause of cancer death in children who live in developed countries.^   1 ^ Acute lymphoblastic leukemia (ALL), the most common cancer of childhood, accounts for approximately 26.8% of all pediatric cancers among children less than 15 years old.^   2 ^ Though the etiology of ALL is mainly unknown, it is recognized that genetic variation plays a critical role in disease development.^3^^,^^4^ The peak rate of childhood ALL happens at age 2 to 5 years, implying that ALL may initiate in utero or during the infant period.^   2 ^ The MTHFR gene is located on short arm of chromosome 1 (1p36.3).^        5 ^ MTHFR protein is a key enzyme in folate metabolism, which catalyzes the irreversible conversion of 5, 10-methylenetetrahydrofolate (5, 10-MTHF) to 5-Methyl THF. 5-Methyl THF is the main circulatory form of folate and provides methyl group for homocysteine methylation and conversion to methionine. It is also involved in formation of S-adenosylmethionine (SAM), the universal donor of one carbon unit for the methylation of different substrates.^6^^-^^8^ Two common polymorphisms in MTHFR gene have been described: First, C to T transition at nucleotide 677 (C677T, rs1801133), which causes the substitution alanine with valine at codon 222 and decreases in the enzymatic activity and subsequently increases the level of homocysteine and altered distribution of folate.^        9 ^ Second, A to C transition at position 1298 (A1298C, rs1801131), which results in the substitution of glutamate to alanine at codon position 429 and also leads to reduced MTHFR activity.^        10 ^

Several genetic association studies have evaluated the associations between ALL and MTHFR gene polymorphisms in various populations and produced contradictory or inconclusive results.^11^^-^^14^ The present study aimed to find out the possible association among rs1801131 (A1298C), rs1801133 (C677T) and rs13306561 polymorphisms in the MTHFR gene and ALL risk in a sample of the Iranian population.

## SUBJECTS AND METHODS

**Patients**

This case-control study has been done in children diagnosed with ALL (n=100) and control individuals (n=120), who were matched for age and sex in southeast of Iran (Zahedan). The study design and the enrolment procedure have been described previously.^15^^-^^18^ Local ethics committee of Zahedan University of Medical Sciences was approved the project and informed consent was obtained from parents of cases and controls (Ethical code: 6858). DNA was extracted from peripheral whole blood using salting-out method.^19^

**Genotyping**

Genotyping of MTHFR rs1801133 (C677T, Ala222Val) and rs13306561 gene polymorphisms was done by PCR-RFLP method. For rs1801133 variant, the forward and reverse primers were 5′-ACTCTCTCTGCCCAGTCCCTGTG-3′ and 5′-AAGAACTCAGCGAACTCAGCACT-3′, respectively.

In each 0.20 ml PCR reaction tube, 1 µl of genomic DNA (~100 ng/ml), 1 µl of each primer, 10 µl of 2X Prime Taq Premix (Genet Bio, Korea) and 7 µl ddH_2_O were added. The PCR conditions were set as follows: 95˚C for 5 min, 30 cycles of 95˚C for 30s, 64˚C for 30s and 72 ˚C for 30s and a final extension step of 72 ˚C for 10 min. Then, 10 µl of PCR product is digested with HinfI restriction enzyme. The T allele was digested and produced 222-bp and 167-bp fragments, while the C allele was undigested and produced 389-bp product.

For MTHFR rs13306561 polymorphism, a set of forward and reverse primer pairs was 5′-GGCCGAGCGTTCTGAGTC-3′ and 5′-CACTAATCCCGCGAAGGGTGCTCA-3′, respectively. The PCR cycling conditions were the initial denaturation step at 95 °C for 5 min, followed by 30 cycles of 95 °C for 30s, 64 °C for 30s, and 72 °C for 30s with a final extension of 72 °C for 10 min. Ten microliters of the PCR product were digested by HpyF3I restriction enzyme. The C allele was digested and produced 162-bp and 23-bp, while the T allele was undigested (185-bp).

Genotyping of MTHFR rs1801131 (A1298C) polymorphism was done using allele specific polymerase chain reaction (AS-PCR). The primer sequences were as follow: generic primers, 5′-AGACCTTCCTTGCAAATACAT-3′; A allele, 5′-GAAGACTTCAAAGACACTCT-3′; C allele, and 5′-GAAGACTTCAAAGACACTCG-3′.

The beta-2 microglobulin (B2MF: 5′-TGTAAACACTTGGTGCCTGATATAGCTTGA-3′, B2MR: 5′-CATCAGTATCTCAGCAGGTGCCACTAATCT-3′) was used as internal control.

The PCR cycling conditions were 95˚C for 5 min, 33 cycles of 95˚C for 30s, 55˚C for 20s, and 72˚C for 30s, followed by a final extension step for 10 min at 72˚C. The products size were 317-bp for either of the A or C alleles and 574-bp for the internal control.

**Statistical analysis**

Statistical analysis was done using statistical package SPSS 20 software. Data were analyzed by independent sample t-test and χ2 test. The association between MTHFR gene polymorphisms and ALL was estimated by computing the odds ratio (OR) and 95% confidence intervals (95%CI) from logistic regression analyses. Haplotype analysis was performed using SNPStats software.^20^ A p-value less than 0.05 were considered statistically significant.

## Results

 The study group involved 100 ALL patients (58 males, 42 females; age: 6.2 ± 3.8 years) and 120 healthy children (56 males, 64 females; age: 5.8 ± 2.2 years). No significant difference was found between the groups concerning sex and age (p=0.105 and 0.303, respectively).

The genotype and allelic frequencies of MTHFR gene polymorphisms in cases and controls are presented in Table 1. The results for rs1801131 (A1298C) variant showed that AC heterozygous genotype decreased the risk of ALL in comparison with AA homozygous genotype (OR=0.43, 95%CI=0.21-0.90, p=0.037). The minor allele frequency (C allele) was not associated with ALL (OR=1.36, 95%CI=0.93-1.98, p=0.12). Neither the overall Chi-square comparison of cases and control subjects (𝜒2=5.54, p=0.063) nor the logistic regression analysis indicated association between C677T polymorphism and ALL (OR=1.25, 95%CI=0.69-2.23, p=0.552; CT vs. CC).

Both rs13306561 TC and TC + CC genotype decreased the risk of ALL compared to TT genotype (OR=0.32, 95%CI=0.15-0.68, p=0.002 and OR=0.35, 95%CI=0.17-0.70, p=0.003, respectively). On the other hand, C allele decreased the risk of ALL in comparison with T allele (OR=0.42, 95%CI=0.22-0.78, p=0.005). We also analyzed the MTHFR polymorphisms interaction (Table 2). In comparison to the reference MTHFR rs1801131AA/ rs1801133 CC/rs2236242 TT, the AC/CC/TT, AC/CC/TC as well as AA/CC/TC genotypes increased the risk of ALL (OR=3.68, 95%CI=1.26-10.78, p=0.017; OR=4.45, 95%CI=1.11-17.90, p=0.048 and OR=7.71, 95%CI=1.28-46.38, p=0.025, respectively).

Haplotype analysis results are shown in Table 3. The findings showed that ACC haplotypes decreased the risk of ALL compared to CCT haplotypes (rs1801131C/ rs1801133C/ rs13306561T). Table 4 summarizes the association of MTHFR polymorphisms with patients’ clinical data. The findings proposed an association between rs1801131 variant and hemoglobin (Hb) levels (p=0.033). Rs1801133 variant was not associated with any clinical symptoms in the patient group, while rs13306561 polymorphism was associated with white blood cells (WBC) (p=0.004) and cerebrospinal fluid (CSF) involvement (p=0.004).

## Discussion

 ALL is a multifactorial disease influenced by genetic and environmental factors. In the current study, we examined the impact of MTHFR gene polymorphisms on the risk of childhood ALL in a sample of the Iranian population. Our findings indicated that rs1801131 (A1298C) as well as rs13306561 T>C variants in MTHFR significantly decreased the risk of ALL in our population. While, no significant association between the MTHFR C677T (rs1801133) variant and ALL was found.

In agreement with our finding a number of previous studies have found no significant association between MTHFR C677T variant and risk of childhood ALL. ^21^^-^^24^  Whereas, few groups have reported that MTHFR C677T variant decreased^25^ or increased the risk of ALL.^12^ Our results showed the same findings regarding the role of MTHFR A1298C in the decrease of the ALL risk.^12^ In contrast to our findings, no significant association between MTHFR A1298C polymorphism and ALL has been found.^26^^-^^29^ Several investigations proposed that MTHFR A1298C polymorphism increased the risk of ALL.^22^^,^^30^^-^^31^ Recently, a meta-analysis research has been done in a Chinese population and its results suggested that both MTHFR C677T and A1298C polymorphisms may be potential biomarkers for ALL risk.^13^

Overall, a significant decreased association was found between the MTHFR C677T polymorphism and ALL risk. In contrast, C677T and A1298C variants were significantly associated with an increased risk of ALL in hospital-based studies, which included individuals with unknown ethnicity.

Dong et al.^14^ have done a meta-analysis and showed that MTHFR rs1801131 CC genotype polymorphism significantly increased the risk of myeloid leukemia in Asian populations. In addition, Jiang et al.^25^ have also indicated that 677TT genotype decreased the risk of ALL in Caucasians in a meta-analysis study (OR=0.715, 95%CI=0.655- 0.781, p=0.000).

**Table 1:** Genotypic and allelic frequencies of MTHFR gene polymorphisms in childhood ALL and control subjects

**Polymorphisms**	**ALL** **n (%)**	**Control** **n (%)**	**OR (95%CI)**	**p-value**
rs1801131 (A1298C)				
AA	21 (21.0)	18 (15.0)	1.00	-
AC	42 (42.0)	83 (69.2)	0.43 (0.21-0.90)	0.037
CC	37 (37.0)	19 (15.8)	1.67 (0.72-3.86)	0.283
AC + CC	63 (63.0)	101 (84.2)	0.53 (0.26-1.08)	0.103
Allele				
A	84 (42.0)	119 (49.6)	1.00	-
C	116 (58.0)	121 (50.4)	1.36 (0.93-1.98)	0.12
rs1801133 (C677T)				
CC	64 (64.0)	92 (76.7)	1.00	-
CT	36 (36.0)	27 (22.5)	1.25 (0.69-2.23)	0.552
TT	0 (0.00)	1 (0.8)	-	-
Allele				
C	164 (82.0)	211 (87.9)	-	-
T	36 (18.0)	29 (12.1)	1.59 (0.94-2.71)	0.105
rs13306561 T>C				
TT	87 (87.0)	84 (70.0)	1.00	-
TC	11 (11.0)	33 (27.5)	0.32 (0.15-0.68)	0.002
CC	2 (2.0)	3 (2.5)	0.64 (0.11-3.95)	0.680
TC + CC	13 (13.0)	36 (30.0)	0.35 (0.17-0.70)	0.003
Allele				
T	185 (92.5)	201 (83.7)	1.00	-
C	15 (7.5)	39 (16.3)	0.42 (0.22-0.78)	0.005

**Table 2:** Interaction of MTHFR rs1801131, rs1801133 and rs13306561 polymorphisms on childhood ALL risk

**rs1801131**	**rs1801133**	**rs13306561**	**ALL n (%)**	**Control n (%)**	**OR (95%CI)**	**p-value**
AA	CC	TT	7 (5.8)	12 (12.0)	1.00	-
AA	CT	TT	5 (4.2)	6 (6.0)	1.43 (0.32-6.46)	0.712
AC	CT	TT	13 (10.8)	16 (16.0)	1.39 (0.43-4.56)	0.765
AC	CC	TT	43 (35.8)	20 (20.0)	3.68 (1.26-10.78)	0.017
CC	CC	TT	13 (10.8)	22 (22.0)	1.03 (0.32-3.22)	0.967
CC	CT	TT	2 (1.7)	12 (12.0)	0.28 (0.05-1.67)	0.240
AC	CC	TC	13 (10.8)	5 (5.0)	4.45 (1.11-17.90)	0.048
AC	TT	TT	6 (5.0)	1 (1.0)	10.29 (1.02-104.0)	0.073
AA	CC	TC	9 (7.5)	2 (2.0)	7.71 (1.28-46.38)	0.025
CC	CC	TC	4 (3.3)	2 (2.0)	3.43 (0.49-23.79)	0.350
AA	CC	CC	1 (0.8)	0 (0.0)	-	-
AA	CT	TC	1 (0.8)	1 (1.0)	-	-
AC	TT	TT	1 (0.8)	0 (0.0)	-	-
AC	CC	CC	2 (1.7)	0 (0.0)	-	-
CC	CC	CC	0 (0.0)	1 (1.00)	-	-

**Table 3:** Haplotype frequencies in childhood ALL and control subjects

**rs1801131**	**rs1801133**	**rs13306561**	**ALL (frequency)**	**Control (frequency)**	**OR (95%CI)**	**p-value**
C	C	T	0.4061	0.4431	1.00	-
A	C	T	0.3197	0.3081	0.83 (0.48-1.45)	0.520
C	T	T	0.0479	0.0917	1.78 (0.54-5.85)	0.340
A	C	C	0.1032	0.0299	0.22 (0.07-0.68)	0.009
A	T	T	0.0639	0.0820	1.12 (0.38-3.24)	0.841
C	C	C	0.0502	0.0389	0.66 (0.18-2.38)	0.520
C	T	C	0.0000	0.0062	-	-
A	T	C	0.0091	0.0000	-	-

**Table 4:** Association of MTHFR gene polymorphisms with demographic and clinical features of patients

**Factors**	**rs1801131**			**p-value**	**rs1801133**			**p-value**	**rs13306561**			**p-value**
	**AA**	**AC**	**CC**		**CC**	**CT**	**TT**		**TT**	**TC**	**CC**	
Sex				0.437				0.435				0.337
Male	10	24	24		38	20	-		51	5	2	
Female	11	18	13		26	16	-		36	6	0	
Age at diagnosis (Year)	6.4 ± 4.1	6.7 ± 3.9	5.6 ± 3.4	0.438	6.6 ± 3.6	5.5 ± 4.0	-	0.160	6.4 ± 3.9	4.7 ± 2.3	7.0 ± 4.2	0.337
WBC (×10^6^/mL)	30.9 ± 36.8	25.9 ± 42.2	44.5 ± 63.4	0.268	34.3 ± 54.4	33.8 ± 44.5	-	0.962	32.4 ± 50.2	24.8 ± 22.2	149.5 ± 43.1	0.004
Hemoglobin (g/dL)	6.9 ± 2.1	7.1 ± 2.8	8.4 ± 2.0	0.033	7.9 ± 2.4	6.9 ± 2.4	-	0.062	7.5 ± 2.4	7.6 ± 2.5	8.9 ± 1.3	0.728
Platelet (×10^6^/mL)	62.4 ± 56.7	55.2 ± 38.6	63.3 ± 54.5	0.757	65.3 ± 54.7	50.4 ± 34.9	-	0.157	59.2 ± 48.4	70.2 ± 55.5	38.5 ± 38.9	0.687
Organomegally				0.087				0.209				0.232
Positive	17	29	34		50	30	-		68	10	2	
Negative	3	11	3		13	4	-		17	0	0	
Lymph adenopathy				0.684				0.463				0.290
Positive	11	21	23		35	20	-		46	8	1	
Negative	9	19	14		28	14	-		39	2	1	
Cerebrospinal fluid involvement				0.418				0.118				0.004
Positive	2	1	3		2	4	-		3	2	1	
Negative	18	40	34		61	31	-		83	8	1	

However, no significant association was found between C677T polymorphism and ALL in Asians and others. In another meta-analysis study, Yan et al.^32^ have found an overall association between 677T variant genotypes and reduced childhood ALL risk, though they found no significant association between MTHFR A1298C polymorphism and childhood ALL risk.

In another meta-analysis, it has been showed that the 677T allele was protective, while A1298C was associated with marginal increase in the risk of childhood ALL.^30^ It has been reported that individuals carrying MTHFR 677T allele may have a higher relative risk of pediatric ALL mortality.^33^ In a meta-analysis study, no association has been reported between MTHFR A1298C and C677T polymorphisms and pediatric ALL.^29^ Recently, it has also been shown that the risk of ALL development in children with MTHFR 677 TT genotype decreased in Asian population.^34^

Atashrazm et al.^35^ investigated the impact of MTHFR C677T and A1298C polymorphisms and the risk of ALL in patients referred to the Iranian Blood Transfusion Organization (Tehran, Iran). In agreement with our finding, they found no significant association between MTHFR C677T variant and the risk of childhood ALL. Interestingly, they also found no association between MTHFR A1298C polymorphism and the risk of ALL. It is noteworthy to mention that the genetic background of our population (Baluch and Sistani) was different from their population, which used in their study (patients referred to the Iranian Blood Transfusion Organization, Tehran, Iran who had different genetic background).

Therefore, it can be concluded that the data regarding the impact of MTHFR on childhood ALL are inconsistent. There is no clear reason for the different results in diverse studies. Ethnic, genetic, and/or environmental factors as well as gene-diet interaction may interact in various ways to either decrease or increase the risk of childhood ALL in different areas.

Taken together, our findings showed that MTHFR rs1801131 and rs13306561 polymorphisms decreased the risk of ALL in a sample of Iranian population. To further confirm these findings, larger sample sizes with diverse ethnicities are required.

## CONCLUSION

 The results of the current case-control study showed an association between MTHFR rs1801131 and rs13306561 polymorphisms and risk of ALL in a sample of Iranian population.
